# Evolution intra-hospitalière et à trois mois des thromboses veineuses cérébrales auprès du service de neurologie de Befelatanana, Madagascar: une étude de cohorte rétrospective

**DOI:** 10.11604/pamj.2022.42.93.29166

**Published:** 2022-06-03

**Authors:** Lala Andriamasinavalona Rajaonarison, Nomena Finiavana Rasaholiarison, Naliniaina Robert Randrianantoandro, Manitra Niaina Rabeony, Julien Razafimahefa, Noël Zodaly, Alain Djacoba Tehindrazanarivelo

**Affiliations:** 1 Faculté de Médecine, Université d’Antsiranana, Antsiranana, Madagascar,; 2 Faculté de Médecine, Université de Fianarantsoa, Fianarantsoa, Madagascar,; 3 Faculté de Médecine, Université d´Antananarivo, Antananarivo, Madagascar

**Keywords:** Anticoagulation, Madagascar, mortalité, thrombose veineuse cérébrale, Anticoagulation, Madagascar, mortality, cerebral venous thrombosis

## Abstract

Le pronostic des thromboses veineuses cérébrales (TVC) est bien meilleur que celui des infarctus cérébraux d´origine artérielle. Nos objectifs étaient de décrire l´évolution intra-hospitalière et à trois mois des TVC auprès du Service de Neurologie de Befelatanana ainsi que la place de l´anticoagulation dans les TVC sans et avec suffusion hémorragique. Nous avons mené une étude de cohorte rétrospective des patients ayant eu une TVC du 1^er^ janvier 2014 au 31 décembre 2019 (72 mois). Leurs caractéristiques cliniques, leurs évolutions intra-hospitalière et après 3 mois ont été collectés. Les données ont été analysées avec le logiciel R en comparant les patients ayant une TVC avec et sans suffusion hémorragique avec un p significatif ≤ 0,05. Nous avons enregistré 21/4227 (0,49%) de cas de TVC dont onze patients (52,38%) ont eu une TVC avec suffusion hémorragique. L´âge moyen était de 38,05 ans. La tranche d´âge de 18 à 34 ans (47,62%) était plus représentée. Le genre féminin prédominait dans 76,19% (n=16). A l´admission, le NIHSS < 10 était de 85,71% (n=18) et le mRS < 3 était de 61,90% (n=13). Tous les patients étaient sous anticoagulation. A la sortie de l´hôpital, le NIHSS < 10 était stable (90,47% (n=19)) avec une augmentation de patient avec un mRS < 3 (85,71% (n=18)) dont 3 étaient du groupe avec suffusion hémorragique. Le séjour hospitalier moyen était de 16,04 jours. Un patient du groupe sans suffusion hémorragique décédait pendant l´hospitalisation. A trois mois de leur sortie, seul 9 patients étaient joignables. Leur état neurologique s´améliorait (NIHSS < 10 dans 100% (n=9), mRS à 0 dans 88,89% (n=8)). Aucune différence statistiquement significative n´était trouvée entre les deux groupes en terme de handicap (p=0,757) avec un RR à 0.91 IC [0.04; 6.55] et de décès (p=0,282) (0 décès dans un groupe) entre les deux groupes mis et non mis sous anticoagulant. La mortalité et le handicap liés à la TVC anticoagulée au cours de son évolution sont très faibles. L´accessibilité à un angioscanner cérébral à faible coût permettra une meilleure prise en charge des TVC dans notre service de Neurologie.

## Abstract (en)

The prognosis of cerebral venous thrombosis (CVT) is much better than that of cerebral artery (CAI) infarct. The purpose of this study is to describe intra-hospital and three-month outcomes of patients with CVT in the Department of Neurology of Befelatanana as well as the role of anticoagulants in the management of patients with CVT, without and with haemorrhagic suffusion. We conducted a retrospective cohort study of patients with CVT from January 01, 2014 to December 31, 2019 (72 months). Patients´ clinical characteristics and their intra-hospital and three-month outcomes data were collected. Data were analyzed using the R software, by comparing data of patients with CVT, with and without haemorrhagic suffusion and with a significant p ≤ 0.05. We recorded 21/4227 (0.49%) cases of CVT, of whom 11 (52.38%) had CVT with haemorrhagic suffusion. The average age of patients was 38.05 years. The age group 18-34 years (47.62%) was most represented. Women were the predominant gender (76.19%; n=16). On admission, NIHSS score < 10 was found in 85.71% of patients (n=18) and mRS score < 3 was found in 61.90% of patients (n=13). All patients were receiving anticoagulants. Upon discharge from hospital, NIHSS score < 10 was stable (90.47% (n=19)) with an increase in patient with mRS score < 3 (85.71% (n=18)), of whom 3 were in the haemorrhagic suffusion group. The average hospital stay was 16.04 days. One patient in the group without haemorrhagic suffusion died during hospitalisation. At 3 months after discharge, only 9 patients were reachable. Their neurological status improved (NIHSS score < 10 in 100% (n=9) of patients, mRS score= 0 in 88.89% (n=8) of patients). No statistically significant differences were found between the two groups in terms of disability (p=0.757), with a relative risk (RR) of 0.91 CI [0.04; 6.55] and of death (p=0.282) (0 deaths in a group) between the two groups receiving and not receiving anticoagulants. Mortality and disability in patients receiving anticoagulants during the evolution of CVT are very low. The availability of a low-cost brain CT angiography could allow better management of CVT in our Neurology Department.

## Introduction

La thrombose veineuse cérébrale (TVC) est une occlusion des veines des sinus duraux et/ou associée à des veines cérébrales. Elle est à l´origine d´une souffrance du parenchyme cérébral [1]. Elle représente 0.5 à 3% des accidents vasculaires cérébraux [2,3]. L´imagerie cérébrale met en évidence la présence du thrombus à l´intérieur de la veine qui en cas d´hyperpression donne la suffusion hémorragique. Elle explique la symptomatologie déficitaire de cette pathologie [4-6]. Actuellement, c´est la seule forme d´hémorragie intra parenchymateuse secondaire qui se traite par la mise sous anticoagulation à dose efficace. Paradoxalement, l´anticoagulation n´aggrave pas l´étendue du saignement [7,8] contrastant avec l´absence de recommandation des sociétés savantes surtout pour les formes compliquées d´hémorragie cérébrale [9]. Cette réalité est due à l´insuffisance des études portant sur ce sujet [10,11]. Dans l´optique d´élargir cette épidémiologie mondiale, nous avons réalisé cette étude visant à décrire l´évolution intra-hospitalière et à 3 mois des patients diagnostiqués comme ayant une thrombose veineuse cérébrale sans et avec suffusion hémorragique, dans le Service de Neurologie du Centre Hospitalo-Universitaire Joseph Raseta Befelatanana (CHU JRB). L´objectif secondaire était de rapporter les caractéristiques clinico-radiologiques des thromboses veineuses cérébrales et d´estimer le risque de survenue d´handicap ou de mortalité sous anticoagulation devant TVC avec suffusion hémorragique.

## Méthodes

**Conception et contexte de l´étude:** nous avons mené une étude de cohorte rétrospective se déroulant sur une période de 72 mois du 1^er^ janvier 2014 jusqu´au 31 décembre 2019 au sein du service de Neurologie du CHU JRB Antananarivo Madagascar.

**Population d´étude**: les participants étaient sélectionnés dans le registre des patients hospitalisés durant la période d´étude. Nous avons inclus tout individu diagnostiqué d´une TVC confirmée par une imagerie cérébrale avec injection (scanner ou une imagerie par résonnance magnétique) et réalisation de fenêtre veineuse. Selon le résultat de l´imagerie, la population a été divisée en groupe I (patients sans suffusion hémorragique) et groupe II (patients avec suffusion hémorragique). Les dossiers incomplets ont été exclus.

**Recueille des données**: les données ont été recueillies à l´aide d´un questionnaire validé par l´équipe de neurologie. Puis les patients étaient revus systématiquement à trois mois du traitement avec une réévaluation neurologique de son état.

**Définition des variables**: nous avons étudié comme variable: la fréquence absolue que relative (par rapport au genre, âge et type d´AVC); la démographie (âge et le genre); les symptômes cliniques d´appel (déficit neurologique, hypertension intracrâniennes, crises épileptiques); le début des symptômes (aiguë, subaiguë ou chronique); l´état neurologique à l´admission évalué par le NIHSS (*National Institute of Health Stroke Scale*), le score de Glasgow et le mRS (*modified Rankin Scale*). Le résultat de l´examen radiologique nous a permis de diviser les patients avec TVC en deux groupes: groupe I (patients sans suffusion hémorragique) et groupe II (patients avec suffusion hémorragique). Nous avons décrit la localisation du thrombus. Nous avons décrit l´évolution hospitalière et à trois des patients en se basant sur l´évaluation des scores neurologiques, le décès et la durée d´hospitalisation. L´anticoagulation était basé sur une héparine à bas poids moléculaire (HBPM) avec relais par du Fluindione et un INR cible entre 2 à 3 pendant 3 mois ou un relais avec du Rivaroxaban pendant 3 mois.

**Analyse statistique:** pour le traitement des données et les tests statistiques, nous avons utilisé le logiciel R et l´association des variables selon le test de Fisher avec un seuil de significativité pour une valeur de p <0,05. Les résultats sont représentés en valeur absolue, en pourcentage et en moyenne. Nous avons comparé nos deux groupes de population sous anticoagulation curative. Nous avons établi la valeur de « p » entre les caractéristiques clinico-radiologiques et l´évolution intra-hospitalière et à 3 mois des patients. Dans cette démarche, nous avons estimé le risque relatif (RR) d´aggravation sous anticoagulation du groupe avec suffusion hémorragique en se basant sur le degré d´handicap (évalué par le mRS) à la sortie et le taux de mortalité hospitalière.

**Considération éthique**: les secrets professionnels et médicaux ont été respectés ainsi que l´anonymat des patients en utilisant des codes.

## Résultats

**Caractéristiques socio-démographiques de la population d´étude**: nous avons retenu 21 patients atteints de TVC sur les 4227 hospitalisés soit 0,49% en prévalence hospitalière. Onze patients (52,38%) ont eu une TVC avec suffusion hémorragique. Notre population d´étude représente 1,11% de tous les AVC. L´âge moyen était de 38,05 ans. La tranche d´âge de 18 à 34 ans (47,62%) était la plus représentée. Le genre féminin prédominait dans 76,19% (n=16).

**Caractéristiques cliniques des TVC**: d´un point de vue clinique (Tableau 1), le mode de survenue de la TVC était subaigu dans 61,91% (n=13) des cas, aigu dans 28.57% (n=6) et chronique dans 9.52% (n=2). Les déficits focaux étaient retrouvés chez 18 patients (85.71%). Ils étaient de type moteur dans 57.14% (n=12), sensitif dans 4.76% (n=1) et aphasique dans 23.81% (n=5). Dix de ces patients faisaient partie du groupe avec suffusion hémorragique. Des crises épileptiques étaient notées dans 66.67% (n=14). Elles étaient des crises tonico-cloniques généralisées (n=7), focalisées (n= 3) et focales secondairement généralisées (n=4). Huit de ces patients font partie du groupe avec transformation hémorragique. A l´admission, un score de Glasgow > 13 avec un score NIHSS < 10 étaient prédominant dans 85,71% (n=18) et un mRS < 2 pour 38,09% (n=8). La thrombose intéressait surtout le sinus sagittal supérieur dans 71,43% (n=15). Elle s´est compliquée d´hématome intra-parenchymateuse dans 52,38% (n=11).

**Tableau 1:** caractéristiques et évolution des thromboses veineuses cérébrales vues dans le service de neurologie, Befelatanana, Antananarivo

variables	Total (N = 21)	Sans suffusion hémorragique (N = 10)	Avec suffusion hémorragique (N = 11)	p
**Age**				
Moyen (en année)	37,38	34,2 [12; 52]	41,55 [27; 89]	
<* 64 ans	20 (95,23%)	10 (100%)	10 (91%)	0,328
>** 64 ans	1 (4,76%)	0	1 (9%)	
**Sex ratio**		0,43	0,22	
**Genre**				
Masculin	5 (23,80%)	3 (30%)	2 (18,20%)	0,525
Féminin	16 (76,19%)	7 (70%)	9 (81,80%)	
**Symptomatologies**				
Hypertension intra-crânienne	12 (57,14%)	6 (60%)	6 (54,55%)	0,800
Déficit neurologique	18 (85,71%)	8 (80%)	10 (90,91%)	0,475
Crises épileptiques focales	3 (14,28%)	1 (10%)	2 (18,18%)	
Crises épileptiques généralisées	7 (33,33%)	3 (30%)	4 (36,36%)	0,757
Crises épileptiques focales secondairement généralisées	4 (19,04%)	2 (20%)	2 (18,18%)	
Triade complet	7 (33,33%)	3 (30%)	4 (36,36%)	0,757
**Début symptomes**				
< 48 heures	6 (28,57%)	2 (%)	4 (36,36%)	0,407
> 48 heures	15 (71,42%)	8 (%)	7 (63,59%)	
**Antécédent péri-partum**	10 (47,61%)	6 (60%)	4 (36,36%)	0,278
**Sinus atteint:**				
Sinus sagittal supérieur	15 (71,42%)	6 (60%)	9 (81,82%)	0,269
Sinus transverse	6 (28,57%)	2 (20%)	4 (36,36%)	
Sinus droit	1 (4,76%)	1 (10%)	0	
Sinus caverneux	1 (4,76%)	1 (10%)	0	
Atteinte multiple	2 (9,52%)	0	2 (18,18%)	

* <: inférieur à, **>: supérieur à

**Traitement et issue des patients**: vingt patients (95.24%) ont été mis sous HBPM avec relais par du Fluindione. Seul un patient (4.76%) du groupe I avait reçu du Rivaroxaban. Sous traitement, la durée moyenne d´hospitalisation était de 16.04 jours, avec des extrêmes allant de 5 à 33 jours. Un patient du groupe sans suffusion hémorragique décédait pendant l´hospitalisation. Les symptômes ont connu une régression en moins de 7 jours d´hospitalisation dans 76,19% (n=16) dont 9 patients appartenaient au groupe II. A la sortie de l´hôpital: 95.24 % de nos patients (n = 20) ont un score de Glasgow à 15; 90,48 % (n = 19) ont eu un score de NIHSS < 10 et un score mRS ≤ 3 dans 85.71 % (n = 18). Seuls 9 patients (45%) sont revenus en consultation à 3 mois d´évolution. Tous ont eu un score de Glasgow à 15 avec un score de NIHSS < 10 dont 8 patients (88.89%) ont eu un mRS à 0 (Tableau 1 (suite)). A la sortie de l´hôpital, nous n´avons pas rapporté de différence significative en terme de handicap (p=0,757) avec un RR à 0.91 IC [0.04; 6.55] et de décès (p=0,282) (0 décès dans un groupe) entre les deux groupes de population ayant été mis et non sous anticoagulant (Tableau 1).

**Tableau 1(suite):** caractéristiques et évolution des thromboses veineuses cérébrales vues dans le service de neurologie, Befelatanana, Antananarivo

variables	Total (N = 21)	Sans suffusion hémorragique (N = 10)	Avec suffusion hémorragique (N = 11)	p
**Score de Glasgow à l'entrée**				
8-12	3 (14,28%)	1 (10%)	2 (18.18)	0,592
13-15	18 (85,71%)	9 (90%)	9 (81.82)	
**Score de Glasgow à la sortie**				
< 8	1 (4,76%)	1 (10%)	0	0,282
13-15	20 (95,23%)	9 (90%)	11(100%)	
NIHSS* à l´entrée				
< 10	18 (85,71%)	9 (90%)	9 (81,82)	0,592
> 10	3 (14,28%)	1 (10%)	2 (18,18)	
**NHISS à la sortie**				
< 10	19 (90,47%)	9 (90%)	10(91%)	0,943
>10	1 (4,76%)	0	1(9%)	
mRS** à l´entrée				
0-3	13 (61,90%)	7 (70%)	6 (54,54%)	0,863
4-6	8 (38,09%)	3 (30%)	5 (45,45%)	
mRS** à la sortie				
0-3	18 (85,71%)	9 (90%)	9 (81,82%)	0,757
4-6	3 (14,28%)	1 (10%)	2 (18,18%)	
**Hémorragie intra crâniènne**				
**0**	**5 (23,80%)**	**5 (50%)**	**0**	**0,007**
**1-6**	**16 (76,19%)**	**5 (50%)**	**11 (100%)**	
**Régression des symptomes**				
<7J	16 (76,19%)	7 (70%)	9 (81,82%)	0,525
> 7 J	4 (19,04%)	2 (20%)	2 (18,18%)	
**Mortalité**	1 (4,76%)	1 (10%)	0	0,282
**Complication TAC**	2 (9,52%)	1 (10%)	1 (9%)	0,943
**Evolution à 3 mois**				
Récidive	1 (4,76%)	1 (10%)	0	0,282
Séquelles	0	0	0	
Décès	0	0	0	
mRS				
0-3	9 (42,85%)	3 (30%)	6 (54,54%)	0,133
4-6	0	0	0	

* NIHSS : National Institute of Health Stroke Scale, **mRS : modified Rankin Scale

## Discussion

A travers notre étude, nous avons voulus décrire l´évolution intra-hospitalière et à trois mois des TVC auprès du Service de Neurologie de Befelatanana ainsi que la place de l´anticoagulation dans les TVC sans et avec suffusion hémorragique. Nous avons enregistré 21 cas (0,49%) de TVC dont onze patients (52,38%) ont eu une suffusion hémorragique. L´âge moyen était de 38,05 ans avec une prédominance du genre féminin dans 76,19% (n=16). La TVC demeure à ce jour un AVC rare (0.5 à 3%) [2,3]. Elle touche surtout la population jeune [4] du genre féminin en rapport avec des facteurs de risque sexo-spécifiques tels que la prise de contraceptive orale, la gravido-puerpéralité [12]. La survenue des symptômes en mode subaiguë prédominait dans 61,91%. Nos patients se présentaient avec un tableau déficitaire focal 85,71% (n=18) de type moteur (n=12) surtout. Arabia B *et al*. [13] rapportaient majoritairement un mode de survenue aigu puis subaigu. Cette différence entre les séries peut être expliquée par les difficultés d´accès aux structures sanitaires dans notre cas. Le type de déficit varie selon la topographie et de l´extension de la thrombose [14]. Les déficits moteurs sont plus fréquents chez les patients avec des TVC au niveau du sinus sagittal supérieur, des veines corticales et devant l´atteinte du système veineux profond cérébral. Notre étude rapportait 66,67% (n=14) de crise épileptique de type généralisée tonoco-clonique dans 33,33% (n=7). Huit de ces patients font partie du groupe avec transformation hémorragique. La crise convulsive est l´un des symptômes les plus fréquemment rencontrés dans la TVC. Elle est présente dans environ 40% des cas [15]. Ceci peut s´expliquer par la proportion plus importante des patients présentant une suffusion hémorragique avec un hématome sus-tentoriel. Les crises épileptiques sont en rapport avec des modifications locales à type d´ischémie ou d´hémorragie [16].

Notre étude rapportait que 18 patients (85,71%) avaient un score de Glasgow > 13 avec un score NIHSS < 10. Le score de Rankin < 2 à l´entrée était rapporté pour 8 patients soit 38,09%. Dans l´étude de Gunes *et al*. [17], tous les patients (100%) avaient un score de Glasgow > 13 à l´admission. Le score de Rankin < 2 était rapporté chez 74,6% des patients. Cette différence s´explique du fait que la majorité de nos patients était diagnostiqué à un stade subaigu de leur pathologie. Des complications liées au thrombus sont déjà présents au moment du diagnostic. A l´imagerie, la TVC se localisait au niveau du sinus sagittal supérieur dans 71,43% (n= 15) des cas. Une suffusion hémorragique était rapportée dans 52,38% (n=11). Le sinus sagittal supérieur est un site de drainage important [18]. Ceci explique qu´il soit la localisation de thrombose la plus fréquemment rencontrée dans notre étude. Nos patients sont venus en consultation à un stade avancé de leur maladie expliquant la présence de saignement car l´évolution d´une TVC vers une suffusion hémorragique est tardive [6].

Sous anticoagulation, les patients étaient en bon état neurologique dans les deux groupes d´étude à leur sortie (score de Glasgow à 15 (n=20), NIHSS < 10 (n=19) et mRS ≤ 3 (n=18)). Un décès était rapporté dans le groupe de TVC sans suffusion hémorragique. Le but du traitement anticoagulant dans la TVC est d´empêcher la croissance du thrombus, faciliter la recanalisation et prévenir la survenue d´autres thromboses (embolie pulmonaire ou thrombose veineuse profonde) [19]. Il est de pratique actuelle que même en présence de lésion hémorragique. Et il n´y avait pas pour autant d´augmentation de l´incidence des hémorragies intracrâniennes [20]. Une méta-analyse de ces deux essais a montré une baisse de la morbi-mortalité chez les patients anticoagulés [20,21]. Sur les 9 patients (45%) revue à 3 mois d´évolution, tous ont un score de Glasgow à 15, avec un score de NIHSS < 10 et 8 patients (88.89%) ont eu mRS à 0. Ce qui démontre que l´efficacité thérapeutique est évaluée sur l´amélioration de l´état clinico-neurologique du patient [22]. Cette étude nous a permis de connaitre l´épidémiologie de la thrombose veineuse cérébrale auprès de notre centre de référence en Neurologie et d´évaluer notre expérience sur l´usage d´une anticoagulation efficace dans les TVC avec transformation hémorragique. Notre faible taux de population d´étude et l´accessibilité limité aux explorations paracliniques surtout à une imagerie de contrôle à trois mois nous a limité dans l´interprétation de nos résultats et ne permet pas une généralisation des résultats.

## Conclusion

La thrombose veineuse cérébrale reste une pathologie rare intéressant surtout les sujets jeunes du genre féminin. Le déficit neurologique focal associé à une crise épileptique évoque le tableau clinique. Le diagnostic est posé par une imagerie cérébrale avec injection (TDM / IRM) qui met en évidence aussi d´éventuelle complication comme une suffusion hémorragique. L´anticoagulation reste le traitement étiologique et efficace de cette pathologie même en présence de suffusion hémorragique. Cette étude nous a permis d´évaluer notre expérience sur l´usage n´aggrave pas l´état clinique du patient. Et de relater les bénéfices pour les patients en termes de récupération fonctionnelle que de la survie.

### Etat des connaissances sur le sujet

- La TVC est une pathologie rare;
- Peu d´études démontrent l´efficacité de l´anticoagulation même en cas de transformation hémorragique de la TVC.

### Contribution de notre étude à la connaissance

- Notre étude a permis d´élargir notre base de données épidémiologiques exploitables sur la TVC;
- Elle apporte une comparaison objective (basée sur les scores neurologiques) de l´évolution sous anticoagulation des cas de TVC sans et avec transformation hémorragique; la mortalité n´augmente pas significativement ni le handicap même si on fait une anticoagulation des patients avec transformation hémorragique de leur TVC.

## Remerciements

Remerciement à toute l´équipe de neurologie du Service de Neurologie du Centre Hospitalo-Universitaire Joseph Raseta Befelatanana.

## References

[1] Bushnell C, Saposnik G (2014). Evaluation and management of cerebral venous thrombosis. Continuum (Minneap Minn).

[2] Kernan WN, Ovbiagele B, Black HR, Bravata DM, Chimowitz MI, Ezekowitz MD (2014). Guidelines for the prevention of stroke in patients with stroke and transient ischemic attack: a guideline for healthcare professionals from American Heart Association/American Stroke Association. Stroke.

[3] Ferro JM, Canhão P (2014). Cerebral venous sinus thrombosis: update on diagnosis and management. Curr Cardiol Rep.

[4] Ferro JM, Canhão P, Stam J, Bousser MG, Barinagarrementeria F, ISCVT Investigators (2004). Prognosis of cerebral vein and dural sinus thrombosis: results of the International Study on Cerebral Vein and Dural Sinus Thrombosis (ISCVT). Stroke.

[5] Piazza G (2012). Cerebral venous thrombosis. Circulation.

[6] Girot M, Ferro JM, Canhão P, Stam J, Bousser MG, Barinagarrementeria F (2007). Predictors of outcome in patients with cerebral venous thrombosis and intracerebral hemorrhage. Stroke.

[7] Guenther G, Arauz A (2011). Cerebral venous thrombosis: a diagnostic and treatment update. Neurologia.

[8] Coutinho JM, de Bruijn SF, deVeber G, Stam J (2012). Anticoagulation for cerebral venous sinus thrombosis. Stroke.

[9] Einhäupl K, Bousser MG, de Bruijn SF, Ferro JM, Martinelli I, Masuhr F (2006). EFNS guideline on the treatment of cerebral venous and sinus thrombosis. Eur J Neurol.

[10] Ndiaye M, Gueye M, Mauferon JB, Ndiaye IP, Kaboré J, Koné S (1987). Les thrombophlébites cérébrales à Dakar: à propos de 54 cas. Dakar Med.

[11] Konin C, Adoh M, Kramoh E, Hauhouot-Attoungbre ML, Amani A, N´Guetta R (2005). Thrombose veineuse cérébrale et du membre inférieur gauche compliquant un déficit en protéine C et une contraception orale. Cardiol Trop.

[12] Rosenstingl S, Ruivard M, Melon E, Schaeffer A, Gouault-Heilmann M (2002). Thrombophlébite cérébrale: étude retrospective de vingt-sept cas. Rev Méd Interne.

[13] Boudjelthia Arbia F, Hatri A, Maamar SA, Hebri ST, Belhadj N (2018). Thrombose veineuse cérébrale: expérience de notre service (à propos de 44 cas). JMV.

[14] Biousse V, Ameri A, Bousser MG (1999). Isolated intracranial hypertension as the only sign of cerebral venous thrombosis. Neurology.

[15] Dentali F, Poli D, Scoditti U, Di Minno MND, De Stefano V, Siragusa S (2012). Long term outcomes of patients with cerebral vein thrombosis: a multicenter study. J Thromb Haemost.

[16] Kalita J, Chandra S, Kumar B, Bansal V, Misra UK (2016). Cerebral venous sinus thrombosis from a Tertiary Care Teaching Hospital in India. Neurologist.

[17] Ueda K, Nakase H, Miyamoto K, Otsuka H, Sakaki T (2000). Impact of anatomical difference of the cerebral venous system on microcirculation in a gerbil superior sagittal sinus occlusion model. Acta Neurochir Wien.

[18] Gunes HN, Cokal BG, Guler SK, Yoldas TK, Malkan UY, Demircan CS (2016). Clinical associations, biological risk factors and outcomes of cerebral venous sinus thrombosis. J Int Med Res.

[19] Crawford SC, Digre KB, Palmer CA, Bell DA, Osborn AG (1995). Thrombosis of the deep venous drainage of the brain in adults. Arch Neurol.

[20] Kumar S, Rajshekher G, Reddy CR, Venkateswarlu J, Prabhakar S (2010). Intrasinus thrombolysis in cerebral venous sinus thrombosis: Single-center experience in 19 patients. Neurol India.

[21] de Bruijn SF, Stam J (1999). Randomized Placebo-Controlled Trial of Anticoagulant Treatment With Low-Molecular-Weight Heparin for Cerebral Sinus Thrombosis. Stroke.

[22] Lee DJ, Ahmadpour A, Binyamin T, Dahlin BC, Shahlaie K, Waldau B (2017). Management and outcome of spontaneous cerebral venous sinus thrombosis in a 5-year consecutive single-institution cohort. J Neurointerv Surg.

